# Supplementary material for: The localization of PHRAGMOPLAST ORIENTING KINESIN1 at the division site depends on the microtubule-binding proteins TANGLED1 and AUXIN-INDUCED IN ROOT CULTURES9 in Arabidopsis
Source: Plant Cell. 2022 Aug 25;34(11):4583–99. doi: 10.1093/plcell/koac266 (PMC9614452; doi:10.1093/plcell/koac266)
Supplement: koac266_Supplementary_Data [file koac266_supplementary_data.zip › koac266_Supplementary_Data/TPC2022RA00398DR1_Supplemental_Figures_1_8_Supplemental_Table_1.pdf]

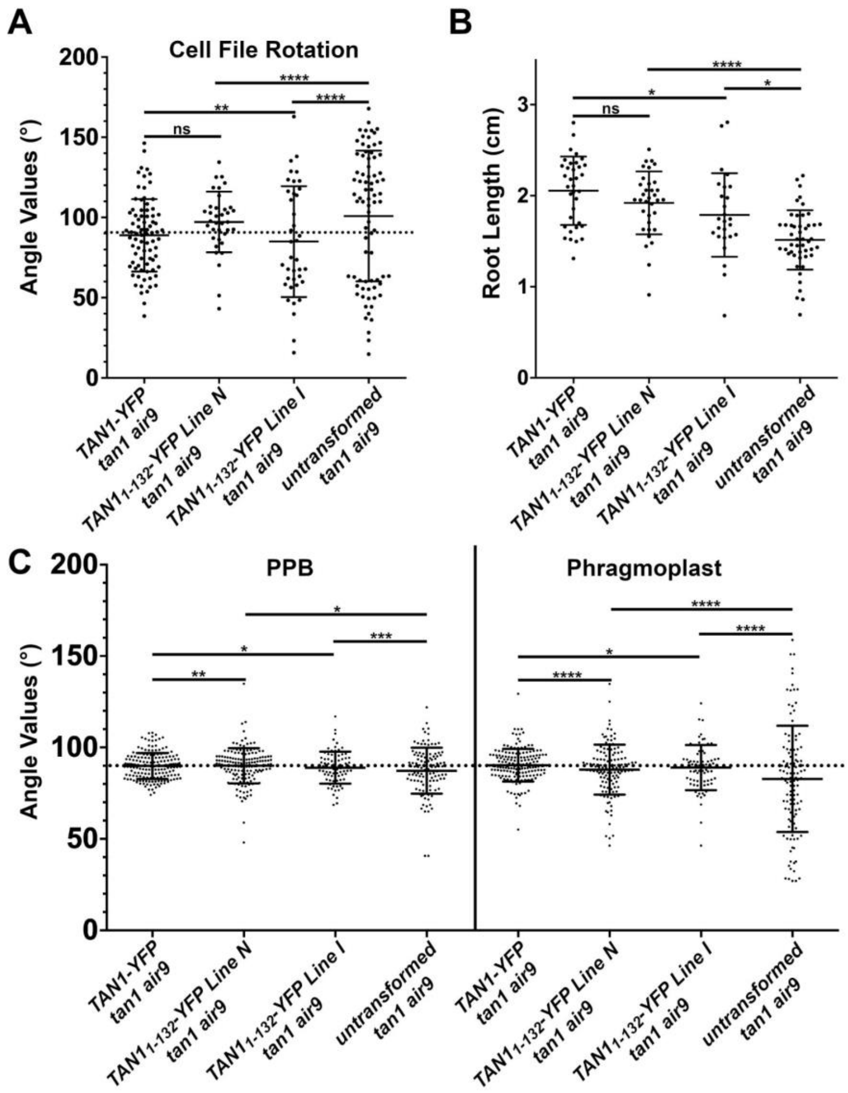

Supplemental Figure S1. *p35S:TAN1*$_{1-132}$*-YFP tan1 air9* lines show significant rescue compared to untransformed *tan1 air9* double mutants. A) Cell file rotation angles of *tan1 air9* double mutants expressing *p35S:TAN1-YFP* (left), two *p35S:TAN1*$_{1-132}$*-YFP* transgenic lines designated as line N (center left) and line I (center right) and untransformed *tan1 air9* plants (right) n > 6 plants for each genotype. Angle variances were compared with Levene's test. B) Root length measurements from 8 days after stratification of *tan1 air9* double mutants expressing p*35S:TAN1-YFP* (left), two *p35S:TAN1*$_{1-132}$*-YFP* transgenic lines (middle) and untransformed plants (right), n > 13 plants for each genotype, compared by two-tailed t-test with Welch's correction. C) PPB and phragmoplast angle measurements in dividing root cells of *tan1 air9* double mutants expressing p*35S:TAN1-YFP* (left), two *p35S:TAN1*$_{1-132}$*-YFP* transgenic lines (middle) and untransformed plants (right), n > 23 plants of each genotype. Angle variations compared with F-test. ns indicates not significant, * P-value <0.05, ** P-value <0.01, **** P-value <0.0001. Mean and standard deviation is indicated. Supports Figure 2.

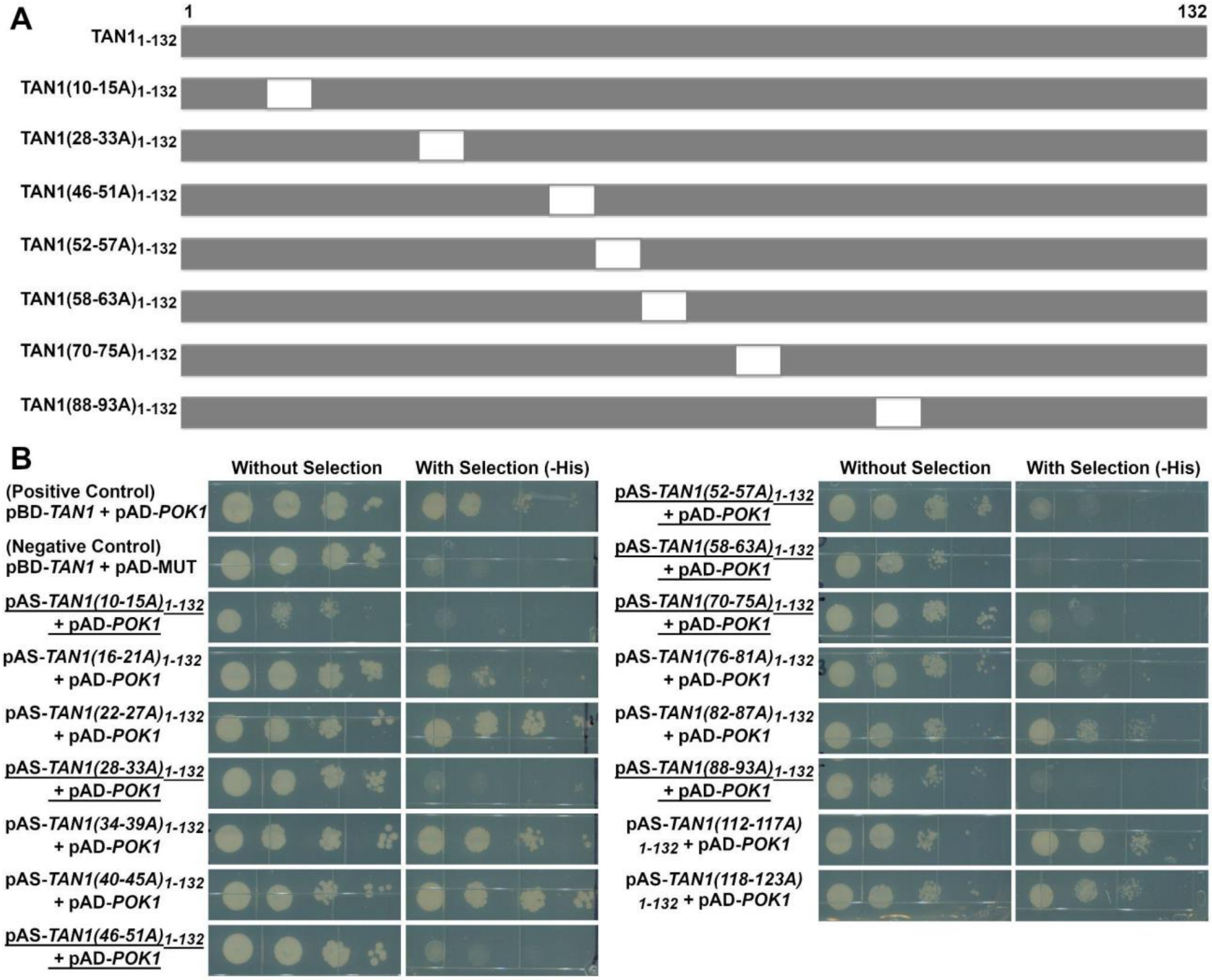

Supplemental Figure S2. Yeast-two-hybrid interactions between POK1 (C-terminal amino acids 1683-2066, as previously described (Müller et al., 2006; Rasmussen et al., 2011; Lipka et al., 2014) **) and TAN1$_{1\text{-}132}$ alanine scanning constructs.** A) Diagram of alanine scanning constructs that showed loss of interaction with POK1 by yeast-two-hybrid. The location of the six alanine substitutions within each TAN1$_{1\text{-}132}$ construct are represented by white boxes. B) Yeast-two-hybrid results of screen for loss of interaction with POK1. Underlined constructs showed loss of interaction with POK1. Alanines 64-69 and 106-111 were not completed and not included in the yeast-two-hybrid. Supports Figure 3 and 4.

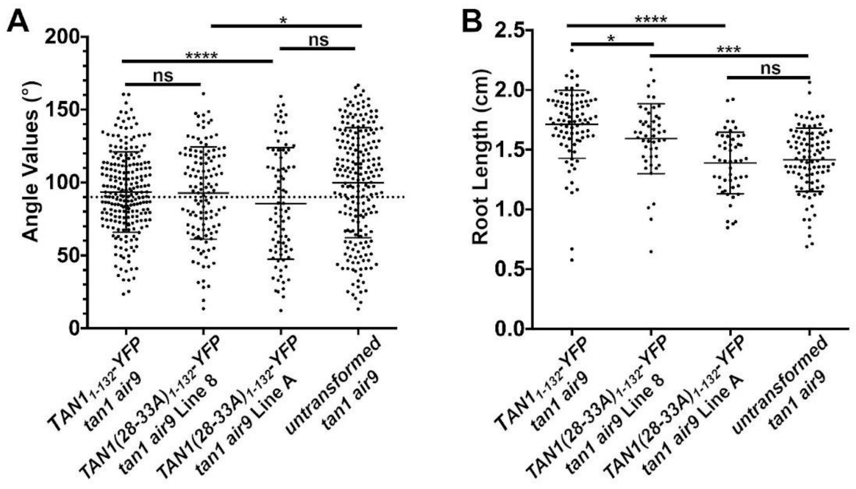

Supplemental Figure S3. *p35S:TAN1(28-33A)$_{1\text{-}132}$-YFP tan1 air9 lines* show variable and incomplete rescue compared to unaltered *p35S:TAN1$_{1\text{-}132}$-YFP tan1 air9.* A) Cell file rotation angles of *tan1 air9* double mutants expressing *p35S:TAN1$_{1\text{-}132}$-YFP* (left) and two *p35S:TAN1(28-33A)$_{1\text{-}132}$-YFP* transgenic lines designated line 8 (second from the left) and line A (second from the right), and untransformed *tan1 air9* double mutants (right). n > 20 plants for each genotype. Variances were compared with Levene's test. B) Root length measurements from 8 days after stratification of *tan1 air9* double mutants expressing *p35S:TAN1$_{1\text{-}132}$-YFP* (left) and two *p35S:TAN1(28-33A)$_{1\text{-}132}$-YFP* transgenic lines (middle), and untransformed *tan1 air9* double mutants (right), n > 33 plants for each genotype, two-tailed t-test with Welch's correction. ns indicates not significant, * P-value <0.05, *** P-value <0.001, **** P-value <0.0001. Supports Figure 4.

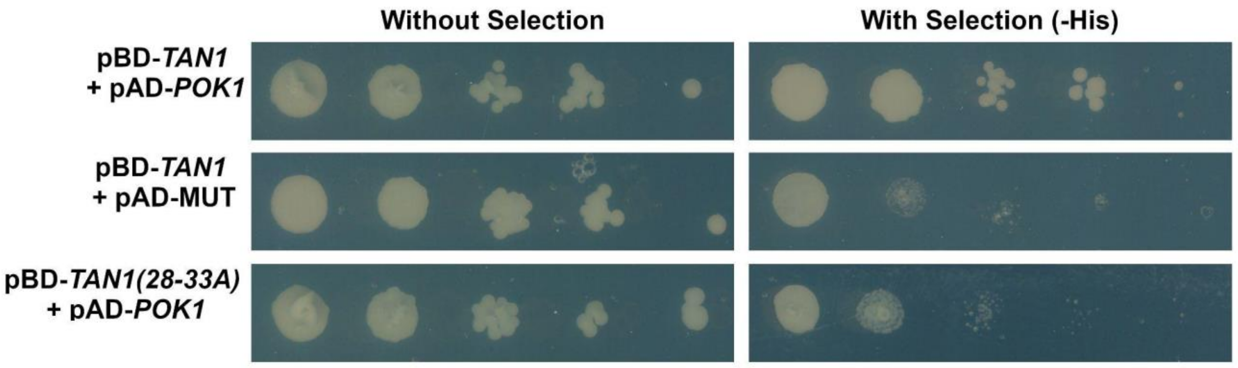

Supplemental Figure S4. Yeast-two-hybrid interactions between TAN1 and POK1 (C-terminal amino acids 1683-2066, as previously described (Müller et al., 2006; Rasmussen et al., 2011; Lipka et al., 2014)**) and TAN1(28-33A).** Full-length TAN1(28-33A) does not interact with POK1 by yeast-two-hybrid. Supports Figure 5.

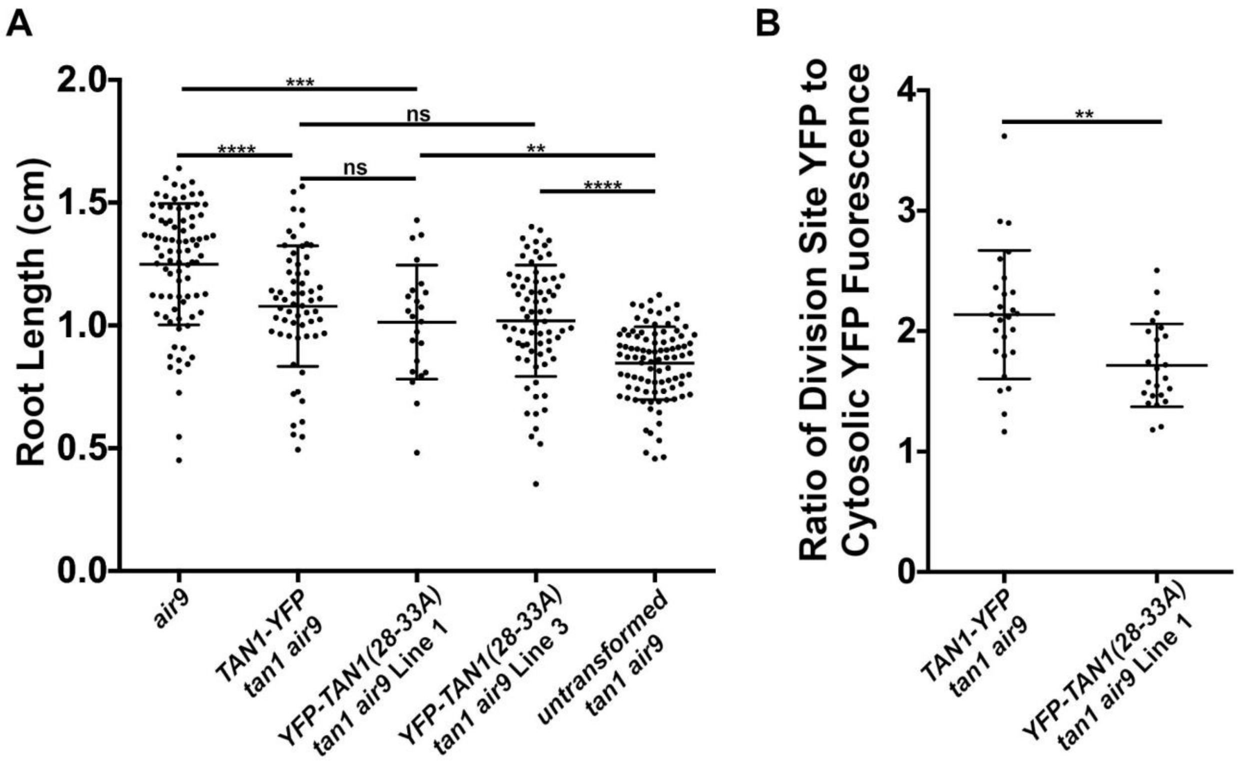

Supplemental Figure S5. ***p35S:YFP-TAN1(28-33A) tan1 air9*** **lines show significant rescue compared to untransformed *tan1 air9,* but less accumulation of YFP-TAN1(28-33A) during telophase.** A) Root length measurements from 8 days after stratification of *air9* single mutants (left), tan*1 air9* double mutants expressing p*35S:TAN1-YFP* (second from the left), two *p35S:YFP-TAN1(28-33A)-YFP* transgenic lines designated as line 1 (center) and line 3 (second from the right), and untransformed plants (right), n > 22 plants for each genotype, compared by two-tailed t-test with Welch's correction. B) Ratio of TAN1-YFP or TAN1(28-33A)-YFP fluorescence at the division site to cytosolic fluorescence from *tan1 air9* plants expressing *p35S:TAN1-YFP* (left) or *p35S:YFP-TAN1(28-33A)* (right) during telophase, n >12 plants for each genotype. Asterisks indicate a significant difference as determined by Mann-Whitney U test. ns indicates not significant, ** P-value <0.01, *** P-value <0.001, **** P-value <0.0001. Note: TAN1-YFP fluorescence measurements are the same as those used for the telophase fluorescence measurements in Figure 5E. Mean and standard deviation is indicated. Supports Figure 5.

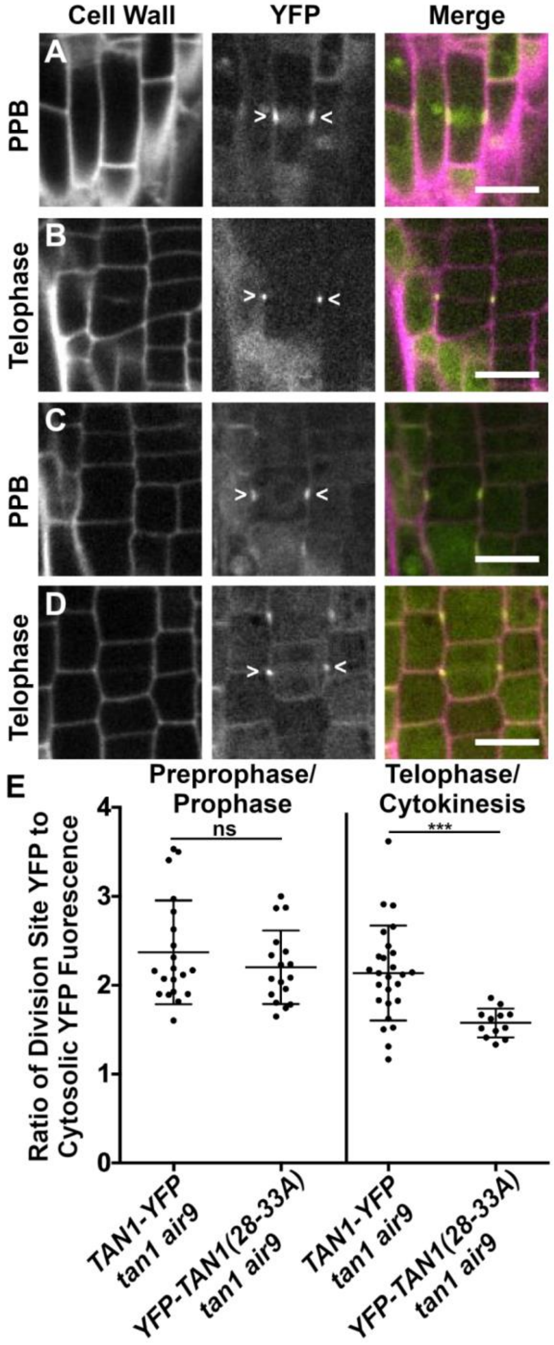

Supplemental Figure S6. YFP-TAN1(28-33A) localizes to the division site in preprophase or prophase and with reduced fluorescence during telophase in *tan1 air9* mutants. Propidium iodide stained *tan1 air9* plants expressing *p35S:TAN1-YFP* in (A) preprophase or prophase (B) telophase or cytokinesis. *tan1 air9* plants expressing *p35S:YFP-TAN1(28-33A)* in (C) preprophase or prophase (D) telophase or cytokinesis. The division site is indicated by arrowheads in the YFP panels. Bars = 10 µm. E) TAN1-YFP or TAN1(28-33A)-YFP ratio of the division site versus cytosolic fluorescence intensity from *tan1 air9* plants expressing *p35S:TAN1-YFP* or *p35S:YFP-TAN1(28-33A)* during preprophase or prophase and telophase or cytokinesis, n >5 plants for each genotype. Ratios compared with Mann-Whitney U test. ns indicates not significant, *** P-value <0.001. Mean and standard deviation is indicated. Supports Figure 6.

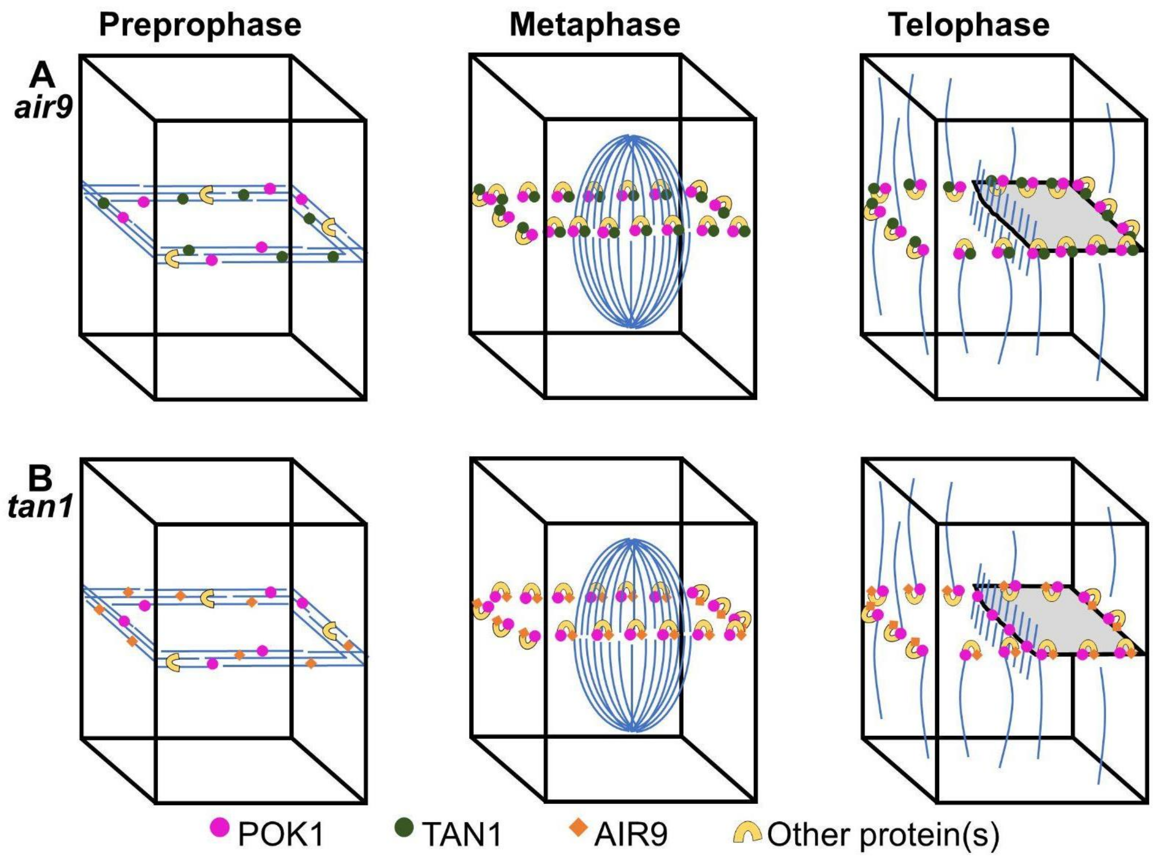

Supplemental Figure S7. A model of POK1 localization in *tan1* and *air9* single mutants. A) In *air9* single mutants TAN1 and POK1 are recruited to the PPB and their interaction with one another and other proteins stabilizes TAN1 and POK1 at the division site. B) In *tan1* single mutants AIR9 and POK1 are recruited to the PPB. POK1 is potentially stabilized at the division site either by interacting directly with AIR9 or another protein recruited to the division site by AIR9. POK1 tends to accumulate in the phragmoplast midline in *tan1* single mutants, which may reflect that POK1 is not as efficiently recruited to the division site in the absence of TAN1. Supports Figure 7.

```
A. thaliana        MVARTPQKQRKVAM-----VVPPLNSDLLKETINKVDKCMERLQELQYTIAGGTKVVSGV
O. sativa          MVARSPDARRSRQTAAAAAAAAALNPALVRETLKKVDRCMARLQELQYTVAGGAKVVSGV
Z. mays            MVARSPNAKPDRQKAAALAAAAALNPALLRETLKKVDRCMARLQELQYTVAGGAKVVSGV
S. bicolor         MVARSPNAKPDRQTAAALAAAAALNPALVRETLKKVDRCMARLQELQYTVAGGAKVVSGV
S. lycopersicum    MVARTPPKLQNKKM-----VVPPLNPILLRETLNKVDKCMARLQELQYTVTGGHKVISGV
B. napus           MVARTPQMQRRVAM-----VVPPLNTELLKETINKVDKCMERLQELQYTIAGGTKVVSGV
                   ****:*             ....**. *:.**::***.** ********::** **:***
```

Supplemental Figure S8. Alignments of amino acids 1-55 of *A. thaliana* TAN1 with TAN1 homologs from other plant species. Amino acids 28-33 of Arabidopsis TAN1 and amino acids that align with them in other plant species are highlighted in green. “*” indicates residues are fully conserved, “:” indicates strong conservation of properties across species, and “.” indicates weak conservation of properties across species. Supports Figure 3 and 4.

Supplemental Table S1. Primers used for cloning and genotyping.

Primer Name	Sequence
ATRP	ATCTCTTAGGAACCAAAACCGGACGCTGT
ATLP	GATCCGTTACGAAAGTGAACACCTTTATC
JL202	CATTTTATAATAACGCTGCGGACATCTAC
AIR9-5RP	TGGATCAGCTGCAACATTATTC
AIR9-5LP	ATTAACATTTTGCAACGCAGG
LBb1.3	ATTTTGCCGATTTCGGAAC
Ds5-4	TACGATAACGGTCGGTACGG
AtTAN 733-CDS Rw	AAATAGAGGGTTCGGAAAAAGAACC
AIR9 gnm7511 R	CCTCCAGTATATGAAGCAACAAAGC
AIR9_cDNA 2230 F	GATGAGGAATATATGTTATCTTTAGATG
Ala_Scan_FOR	GCCGTCACAGATAGATTGGCT
Ala_Scan_Rev	GAAAGCAACCTGACCTACAGG
Ala_02_FOR	GCTGCTGCCGCTGCCGCTGTGCCTCCTCTCAACTCAGAT
Ala_02_Rev	AGCGGCAGCGGCAGCAGCCTGCTTCTGTGGGGTTCT
Ala_03_FOR	GCTGCTGCCGCTGCCGCTGATCTTCTCAAGGAAACGATCAAC
Ala_03_REV	AGCGGCAGCGGCAGCAGCCACCATCGCCACTTTCCT
Ala_04_FOR	GCTGCTGCCGCTGCCGCTATCAACAAGGTTGATAAATGTATGGAA
Ala_04_REV	AGCGGCAGCGGCAGCAGCTGAGTTGAGAGGAGGCACCAC
Ala_05_FOR	GCTGCTGCCGCTGCCGCTTGTATGGAAAGACTGCAAGAGCTA
Ala_05_REV	AGCGGCAGCGGCAGCAGCCGTTTCCTTGAGAAGATCTGAGTT
Ala_06_FOR	GCTGCTGCCGCTGCCGCTGAGCTACAGTACACAATTGCAGGA
Ala_06_REV	AGCGGCAGCGGCAGCAGCTTTATCAACCTTGTTGATCGTTTCCTT
Ala_07_FOR	GCTGCTGCCGCTGCCGCTGCAGGAGGAACCAAAGTTGTC
Ala_07_REV	AGCGGCAGCGGCAGCAGCTTGCAGTCTTTCCATACATTTATCAAC
Ala_08_FOR	GCTGCTGCCGCTGCCGCTGTCTCTGGTGTGAACCTTAGC
Ala_08_REV	AGCGGCAGCGGCAGCAGCAATTGTGTACTGTAGCTCTTGCAG
Ala_09_FOR	GCTGCTGCCGCTGCCGCTAGCCCTCGAAGCACTAGA
Ala_09_REV	AGCGGCAGCGGCAGCAGCAACTTTGGTTCCTCCTGCAAT
Ala_10_FOR	GCTGCTGCCGCTGCCGCTATTTACTTGAAGACTAGTCTTAGATGCAAG
Ala_10_REV	AGCGGCAGCGGCAGCAGCAAGGTTCACACCAGAGACAAC
Ala_12_FOR	GCTGCTGCCGCTGCCGCTACTTTAAGGATCAAGAATGCTACTAATAAG
Ala_12_REV	AGCGGCAGCGGCAGCAGCACTAGTCTTCAAGTAAATTCTAGTGCT
Ala_13_FOR	GCTGCTGCCGCTGCCGCTGCTACTAATAAGAAATCTCCAGTAGGG
Ala_13_REV	AGCGGCAGCGGCAGCAGCTTCTTGCTTGCATCTAAGACTAGT
Ala_14_FOR	GCTGCTGCCGCTGCCGCTCCAGTAGGGAAGTTTCCTGCT
Ala_14_REV	AGCGGCAGCGGCAGCAGCATTCTTGATCCTTAAAGTTTCTTGCTT
Ala_15_FOR	GCTGCTGCCGCTGCCGCTGCTTCCTCACCAGGAGATTGG
Ala_15_REV	AGCGGCAGCGGCAGCAGCAGATTTCTTATTAGTAGCATTCTTGATCCT
Ala_16_FOR	GCTGCTGCCGCTGCCGCTTGGAGGAAAATGTCACTCCCA
Ala_16_REV	AGCGGCAGCGGCAGCAGCAGGAAACTTCCCTACTGGAGA
Ala_17_FOR	GCTGCTGCCGCTGCCGCTCCAGCAATGCTACTAGGAGAG
Ala_17_REV	AGCGGCAGCGGCAGCAGCATCTCCTGGTGAGGAAGCAGG
Ala_19_FOR	GCTGCTGCCGCTGCCGCTTTACAAGCCTCACAGGTCACA
Ala_19_REV	AGCGGCAGCGGCAGCAGCTCCTAGTAGCATTGCTGGGAG
Ala_20_FOR	GCTGCTGCCGCTGCCGCTACAAGAGACATTGTGGACGCC
Ala_20_REV	AGCGGCAGCGGCAGCAGCGATTTCATTTACAGTCTCTCCTAGTAGCAT
NpTANSacIFor	GTATGAGCTCCGGTAGAGTTGAACCAG
NpTANceruleanRev	CCTCGCCCTTGCTCACCATCTTCTATATATATTTTCTTTA

NpTANceruleanFor	TAAAGAAAATATATATAGAAGATGGTGAGCAAGGGCGAGG
CeruleanpEarleyRev	GGCCCGCGGTACCGTCCTTGTACAGCTCGTCCATGC
CeruleanpEarleyFor	GCATGGACGAGCTGTACAAGGACGGTACCGCGGGCC
pEarleyOCSPstIRev	CCATCTGCAGCTGCTGAGCCTCGACAT
AtExon1_1For	CTCAACTCAGATCTTCTCAAGGAAACG
At255AfterStopRev	GCATAGTGGTACCCTCAAATTACACC
