# Supplementary material for: The localization of PHRAGMOPLAST ORIENTING KINESIN1 at the division site depends on the microtubule-binding proteins TANGLED1 and AUXIN-INDUCED IN ROOT CULTURES9 in Arabidopsis
Source: Plant Cell. 2022 Aug 25;34(11):4583–99. doi: 10.1093/plcell/koac266 (PMC9614452; doi:10.1093/plcell/koac266)
Supplement: koac266_Supplementary_Data [file tpc22.00398.PeerReviewReport.pdf › koac266_Supplementary_Data/tpc22.00398.PeerReviewReport.pdf]

Localization of PHRAGMOPLAST ORIENTING KINESIN1 at the division site depends on the microtubule binding proteins TANGLED1 and AUXIN-INDUCED IN ROOT CULTURES9 in Arabidopsis

Alison M. Mills[1], Victoria H. Morris[2,3], Carolyn G Rasmussen

Corresponding author: Carolyn Rasmussen: carolyn.rasmussen@ucr.edu.

Review timeline:

**TPC2021-RA-00375**	Submission received:	April 27, 2021
	1st Decision:	June 5, 2021 *manuscript declined (might reconsider)*
**TPC2022-RA-00398**	Submission received:	April 26, 2022
	1st Decision:	June 27, 2022 *accept with minor revisions*
**TPC2022-RA-00398R1**	1st Revision received:	July 26, 2022
	2nd Decision:	August 8, 2022 *acceptance pending, sent to science editor*
	Final acceptance:	August 22, 2022

REPORT: (The report shows the major requests for revision and author responses. Minor comments for revision and miscellaneous correspondence are not included. The original format may not be reflected in this compilation, but the reviewer comments and author responses are not edited, except to correct minor typographical or spelling errors that could be a source of ambiguity.)

TPC2021-RA-00537 1st Editorial decision – *declined* **June 5, 2021**

Thank you for choosing to send your manuscript entitled "Functional characterization of TANGLED1 interaction with PHRAGMOPLAST ORIENTING KINESIN1 during mitosis in Arabidopsis" for consideration at The Plant Cell. Your submission has been evaluated by members of the editorial board as well as expert reviewers in your field, and we regret to inform you that we are not able to recommend publication of this manuscript in its current form. We have not made this decision lightly. We have had input from multiple scientists, and we have solicited post-review comments as well. Our present policy is to offer streamlined decisions and to not advise on the direction of the work by requesting extensive modifications or substantial additional experiments.

That said, during the post-review consultation session, we agreed that if you could address the major points raised by the reviewers by new experiments, we would welcome a resubmission. This may be treated as a new submission, but we would attempt to use at least some of the same reviewers. Nevertheless, reviewers will be asked to assess as a new manuscript (i.e. are the claims fully supported by the data and do the results presented move the field forward?), and not only whether previous reviewer comments have been addressed.

All the reviewers agreed that the manuscript has the potential to add to the understanding of the mechanism of cell division. However, all reviewers indicated that the conclusions were overstated and that there are major issues that need to be addressed to fully support the conclusions in the manuscript. Some of the key points that we believe need to be addressed are:
1. Investigate the localization of Pok1/MTs during the entire cell cycle in knock-out/knock-down mutants and in lines expressing the TAN deletion and motif mutants to be able to substantiate the claims made in the manuscript.
2. Perform rescue experiments with the native promoter. As reviewer 2 pointed out, the Knolle promoter may not lead to the same levels as the tangled promoter. Furthermore, excess expression due to less degradation may be the reason for rescue of interphase phenotypes.
3. Need to more tightly connect the yeast two-hybrid data with the in planta results.
4. Work on the writing of the manuscript - be sure to tone down statements that are not well supported and give a better rationale for the general approach of the study.

It will be important to convince the editors and reviewers that the major claims made are fully justified by the data presented. This includes careful consideration and explanation of the various controls used in experiments, the extent and manner of replication, and the statistical analyses used. Sampling methods and the nature of "biological replicates" should be described precisely (i.e. different plants, parts of plants, pooled tissue, independent pools of tissue, sampled at different times, etc.), along with a clear description of and rationale for any statistical analyses conducted. The reader should know exactly what was sampled; what forms the basis of the calculation of any means and other statistical variables and parameters reported. This is also necessary to ensure that proper statistical analysis was conducted.

-- Reviewer comments:

Reviewer comments on previously declined manuscript and **author responses:**

Reviewer #1:

TAN1 and POK1 are important components of the division site at the cell cortex and implicated in phragmoplast guidance. Previous work from a different group established independent recruitment of the two proteins in prophase, while maintenance of TAN at the division site was POK-dependent (Lipka et al., 2014). This finding was independently confirmed in the current study and further detailed by mutating the POK-binding site within the TAN amino-terminus, leading to impaired POK-binding, and to reduced persistence of the mutated TAN-variant throughout mitosis and cytokinesis. The authors propose that their findings support a function for TAN in the recruitment of POK1 during telophase and suggest that even little TAN is required for phragmoplast guidance in telophase because the reduction of TAN after metaphase had little effect on phragmoplast guidance (shown in previous work). Furthermore, expressing TAN from the mitosis-specific KNOLLE promoter rescues differentiation defects in the tan air9 mutant, suggesting TAN mitotic functions are sufficient to rescue defects in the differentiation zone. The data were collected using yeast two-hybrid interaction assay, genetic analysis, and confocal imaging.

Point 1. My main concern is that the localization of POK1 has not been evaluated during the entire cell cycle in knock-out/knock-down mutants and in lines expressing the TAN deletion and motif mutants. I think these experiments are mandatory to evaluate/support the claims made in this manuscript.

> **RESPONSE: We crossed the microtubule marker UBQ10:mScarlet-MAP4 into tan1 air9 plants expressing pPOK1:YFP-POK1. This enabled us to examine YFP-POK1 localization to the division site throughout the cell cycle. We found that YFP-POK1 is lost from the division site upon entry into metaphase. This result suggests that POK1 can colocalize with the preprophase band independently of AIR9 and TAN1, but that TAN1 and AIR9 are then required (directly or indirectly) to hold POK1 at the division site once the PPB is disassembled. We also introduced *pTAN:CFP-TAN1, which rescues the double mutant phenotype (rescue information available here* :https://doi.org/10.1101/2021.04.30.442137) and *pTAN:CFP-TAN1(28-33A)* constructs and introduced them into *tan1 air9* double mutants expressing *YFP-POK1* and *mScarlet-MAP4* in order to examine both TAN and POK. We found that the amino acid substitutions in TAN1(28-33A) disrupt, but do not completely abolish, both TAN1(28-33A) and POK1 localization to the division site after PPB disassembly. Based on these data and the assumption that TAN1(28-33A) does not interact with POK1 we hypothesize that there are other protein(s) that mediate TAN1 and POK1 recruitment and maintenance to the division site.**

Point 2: An alternative interpretation of the presented data might be, that TAN plays a minor role in phragmoplast guidance if POK1 is present during telophase. Therefore, authors need to clarify whether POK1 is recruited, but not maintained during the cell cycle, particularly when co-expressed with TAN(132aa) in tan air9 double mutants (experiment shown in Figure 3). It is important to know the frequencies of POK1 in different cell cycle phases to understand whether recruitment during prophase or maintenance of POK1 during mitosis is compromised in the presence of TAN(132) in tan air9 double mutant (or in other genotypes as follows).

> **RESPONSE: We addressed this question by analyzing full length TAN1(28-33A) and POK1 localization in the *tan1 air9* double mutant. We demonstrate that in plants expressing full length TAN1(28-33A) both POK1 and TAN1(28-33A) accumulation at the division site are reduced during metaphase and early telophase. These results are consistent with the hypothesis that TAN1-POK1 interactions are mediated by the 28-33 amino acid motif.**

Point 3. Investigation of POK1 localization in different mutants lead to the conclusion that TAN and AIR9 together were required for POK1 localization during telophase and also implicated a second recruitment round of POK1 during telophase. The current view is that POK1 is recruited once in prophase and maintained at the division site, even in the absence of microtubules. To know what happens to POK1 during the cell cycle, co-expression with a microtubule reporter is critical. Previous work showed that POK1 recruitment to PPBs occurs progressively during prophase because PPBs showed degrees of cytoplasmic POK1 in the absence and presence of POK1 rings in wild-type root meristems (Lipka et al., 2014). In Figure 6, POK1 rings in prophase are shown, but since no microtubule reporter is present, it is unclear how many cells had a PPB but no POK1 ring yet. In the current manuscript, only prophase cells with POK1 rings were counted disregarding prophase cells/PPBs with cytoplasmic POK1. Thus, the quantification needs to be performed in a line co-expressing POK1 and a microtubule reporter and evaluated each cell cycle stage, not just pro- and telophase. In my view, this is important to clarify when exactly POK1 is lost from the division site in the absence of tan air9 if the authors wish to prove that a second recruitment phase exists for POK1 during telophase that this might be TAN AIR9 dependent.

RESPONSE: Thank you for this suggestion. We crossed in the microtubule marker UBQ10:mScarlet-MAP4 into tan1 air9 plants expressing YFP-POK1. We found that YFP-POK1 is lost from the division site upon entry into metaphase. This result suggests that POK1 can colocalize with the preprophase band independently of AIR9 and TAN1, but that either TAN1 or AIR9 are then required to hold POK1 at the division site once the PPB is disassembled. Confocal images of YFP-POK1 localization in wild-type, tan1 air9 double mutants, and tan1 and air9 single mutants were added in Figure 1, and cell counts of YFP-POK1 localization to the division site and phragmoplast midline are now shown in Table 1.

Point 4. Figure 5. The reduction of TAN(28-33aa) at the division site during telophase might reflect the inefficient recruitment of POK1 due to impaired interaction with the TAN mutant. Thus, the partial phenotype rescue might be a result of less efficient POK1 recruitment and the consequent loss of its function in telophase cells. Thus, POK1 localization in this line should clarify the issue.

RESPONSE: We developed *CFP-TAN1* and *CFP-TAN1(28-33A)* constructs and introduced them into *tan1 air9* double mutants expressing *YFP-POK1* and *mScarlet-MAP4* in order to examine the effects of the 28-33 alanine substitutions on POK1 localization to the division site. CFP-TAN1(28-33A) and YFP-POK1 had minor defects in maintenance at the division site during metaphase and early telophase. However, both proteins were present at the division site in 90% of late telophase cells. Figure 5 is now Supplementary Figure 5. CFP-TAN1 and CFP-TAN1(28-33A) images and cell counts of localization during division can be found in the new Figure 6 and Table 2.

Point 5. Thus, one could argue that POK1 recruitment is partially dependent on TAN and AIR9, but there might be other MAPs involved in its recruitment as well since POK1 is present in prophase cells in tan air9 mutants and consistent with the data presented in Figures 3 and 6. POK1 might not be properly recruited in prophase and therefore might be absent from the division site in mitosis/cytokinesis in a subset of cells. POK1 could also be lost during metaphase, which would favor a failure in POK1 maintenance during mitosis or just before telophase, supporting the author's idea of a second POK1 recruitment phase in telophase, but this needs to be clarified.

RESPONSE: We demonstrate that POK1 is lost from the division site after PPB disassembly in the *tan1 air9* double mutant, which suggests that either TAN1 or AIR9 is sufficient for POK1 maintenance at the division site.

Point 6. Based on our observations of CFP-TAN1(28-33A) and YFP-POK1 localization in the *tan1 air9* double mutant with a microtubule marker we hypothesize that POK1 and TAN1 are maintained at the division site via interaction with other protein(s) and that disrupting POK1-TAN1 interaction weakens maintenance of these proteins at the division site.

RESPONSE: Our observation that CFP-TAN1(28-33A) and YFP-POK1 are lost from the division site in 42% of metaphase and 35% of early telophase cells but are present at the division site in 90% of late telophase cells supports the hypothesis that POK1 may be recruited by independent mechanisms during telophase. The large number of late telophase cells with POK1 and TAN1(28-33A) likely accounts for the nearly complete rescue of the *tan1 air9* mutant and supports the hypothesis that TAN1 and POK1 are crucial for phragmoplast guidance during telophase.

Point 7. POK1 localization in the *tan air9* mutant is abolished in only half of the cells, consistent with a slightly milder phenotype compared to complete loss of both POK1 and POK2 in *pok1 pok2* double mutants.

In the *tan1 air9* double mutant, YFP-POK1 was not observed at the division site in cells after metaphase. Instead YFP-POK1 sometimes accumulated in the phragmoplast midline. (Figure 1, Table1). In contrast, YFP-POK1 co-localizes with the PPB (Figure1, Table1).

<u>Reviewer #2:</u>

This manuscript describes further characterization of the function of the N-terminal domain (amino acids 1-132) of TANGLED1 in Arabidopsis thaliana by demonstrating its ability to rescue the mutant phenotype of the tan1 air9 double mutant. The authors further go on to show that a 6-Ala mutation in this N-terminal domain disrupts POK1 binding and reduces the ability of this domain to localize to the cortical division site during telophase and to rescue the double mutant phenotype. Interestingly, the same mutation in the context of the full-length TAN1 protein had reduced localization defects and also was able to better (although not fully) repair the double mutant phenotype. Furthermore, the authors demonstrate that both TAN1 and AIR9 are able to recruit POK1 to the cortical division site and that exclusive expression of TAN1 in dividing cells is sufficient to rescue apparent interphase defects (cell twisting).

This is an interesting manuscript that provides additional insights into the functioning of TANGLED proteins during cell plate organization. The positive identification of the N-terminal 132 amino acids as being sufficient for the majority of TANGLED functions is an important step forward in helping to elucidate the precise mechanisms by which these proteins act. The delineation of protein sequences involved in POK1 binding is also extremely valuable as it allows for a more surgical approach to experiments. The authors take advantage of this with the (28-33A) constructs which provide promising new insights.

Point 1. While I find this approach overall very promising, I think the conclusions presented in the manuscript overreach what can be derived from the experimental data and do not support the broad statement made in the title.

RESPONSE: Thank you, we have significantly revised this manuscript with your comments in mind.

Point 2. My main concern is that there is no direct connection between the yeast two-hybrid results in Supplementary Figure 2 and the in planta experiments in Figures 2-5. Specifically, it is not clear whether the defects detected in the seedlings expressing the 6-Ala mutant are caused by the inability of the mutant protein to bind POK1 (in yeast cells). The replacement of amino acids 28-33 (INKVDK) with AAAAAA could specifically affect the residues that directly bind to POK1, but it could also lead to other, maybe conformational changes that not only prevent efficient binding of POK1 but also of other proteins (also see the next point). This uncertainty makes statements like "preferential localization of TAN1[1-132]-YFP to the division site during telophase is dependent on this interaction with POK1" (line 200; also see the following sentence in line 203, and Discussion line 281) untenable. I think it would be advisable to either tone down the conclusions in this respect, or to provide additional support for the contention that it is specifically the reduced interaction with POK1 that causes the reduced functionality. For example, it might be possible to use some of the other alanine replacement mutants to test their ability to rescue the tan1 air9 double mutant. If these other mutants would show the same defects, then it would be more difficult to argue that they all act through the same novel change in protein interactions.

RESPONSE: We have substantially changed the way that we discuss transgenes, specifying that their interaction has been disrupted in the yeast-two-hybrid system, and discussing the alternative hypothesis that lack of interaction in yeast may not reflect lack of interaction in Arabidopsis in the discussion. "Here we hypothesize that amino acids 28-33 are essential for TAN1 and POK1 interaction in both yeast-two-hybrid and in Arabidopsis. In addition to several reports showing that TAN1 and POK1 interact using the yeast-two-hybrid system (Müller et al. 2006; Rasmussen et al. 2011), bimolecular fluorescence complementation has also been used to show TAN1-POK1 interactions in Arabidopsis protoplasts (Lipka et al. 2014). Alanine substitutions at positions 28-33 of TAN1 may disrupt TAN1-POK1 interaction through misfolding that blocks the POK1 interaction site or by affecting the amino acids that directly mediate POK1 binding. Regardless of the exact mechanism(s) of POK1-TAN1 physical interactions or the possibility that yeast-two-hybrid interactions do not reflect equivalent POK1-TAN1 physical interactions in Arabidopsis, we show that these TAN1 amino acids are involved in mediating TAN1 and POK1 localization to the division site."

Point 3. I find it remarkable that there are so many regions of the N-terminal region of TAN1 that are apparently required for interaction with POK1 (Supplementary Figure 2). This brings up questions on how binding to POK1 is actually occurring and whether all these sequences directly interact with POK1. Obviously, these are very different questions from those asked in this manuscript, but they still impinge on the selected experimental strategy and the interpretation of the data. Specifically, I would have liked to see an explanation for the selection of the (28-33A) mutant for in planta rescue experiments. What sets this particular 6-Ala mutant apart from the others that promised more informative results? Is the disrupted amino acid sequence particularly highly conserved among TANGLED homologs? Or do other experiments implicate these residues in protein-protein interactions? Related to the previous point, is there reason to assume that this particular mutation leads to fewer secondary effects than the others? While there may not be simple, clear-cut answers to these questions, it may be possible to provide some educated guesses by looking at sequence conservation and secondary structure predictions.

RESPONSE: Thank you for this insightful comment. We included more information in the results and discussion about the highly conserved amino acid sequences within TAN1 28-33 (Supplementary Figure 7). In the introduction, we also mention that TAN is an intrinsically disordered protein, so secondary structure predictions may not be reliable. While we agree that this is a very important question, and are in the process of transforming other

transgenes with the disrupted sequences, it takes a significant amount of effort to perform adequate analysis.

Point 4. The experiments with the KNOLLE promoter are quite elegant, but I am not quite sure how much they expand our understanding of TANGLED function. It is already well established that the Arabidopsis TAN promoter is active primarily in dividing tissues (Walker et al 2007) and hence has an expression pattern that closely resembles that of pKN. It should therefore come as no surprise that expression of TAN-YFP from pKN also rescues the mutant phenotype of the tan1 air9 double mutant. In addition, I urge the authors to be careful about their interpretation that "mitotic" TAN protein can rescue "interphase" defects. The separation of these cell cycle phases is not as strict as often assumed. Specifically, there are two aspects worth considering:

(a) pKN is a very strong promoter and can drive high accumulation of mRNA (and presumably protein) during G2 and M phases. This may lead to a high concentration of TAN1 protein in dividing cells.

(b) There does not seem to be a cell cycle-specific degradation signal on TAN1 (unlike KN), so there will still be some protein around during interphase that will get diluted out as the cell grows (and the protein naturally turns over). As a result, we cannot be sure that the rescue activity of the pKN:TAN1-YFP construct occurs only during mitosis. It is quite possible that the TAN1-YFP that is still present early after mitosis is sufficient to set up a relatively normal interphase microtubule array that is then maintained during cell expansion to allow for normal (not twisted) cell elongation.

It would be helpful to look at the microtubule organization in the elongation zone of the different genotypes to see whether they show differences in transverse versus helical arrangements.

RESPONSE: We decided that the pKN:TAN1-YFP rescue data did not lend additional support to our hypotheses about POK1, TAN1, and AIR9 interaction so we removed it from this manuscript. The pKN:TAN1-YFP data has been submitted as its own article and is currently posted on BioRxiv (https://doi.org/10.1101/2021.04.30.442137). We used your very helpful comments to more carefully discuss what the pKN results mean in terms of location (meristematic) instead of cell cycle (mitotic). Further, we demonstrate that TAN1 driven by its native promoter, including pTAN:CFP-TAN1, fully rescues the *tan1 air9* double mutant.

Point 5. I am confused by the measurement of "Cell File Rotation" (Figures 1C, 2D, 4C, 8C, Supplementary Figures 1A, 5A). From the description in the methods section, it seems that the indicated angles are those of the transverse (i.e. cross) walls, i.e. of walls were introduced during cell divisions in the meristematic zone. The term Cell File Rotation, however, clearly talks about the orientation of the entire cell, which would best be captured by measuring the longitudinal walls of the cells. This latter measurement would also directly reflect the strong phenotype seem in Figure 1B, 2B, etc. In fact, the angle of the cross wall may deviate greatly from 90{degree sign} but the cell files can still appear perfectly straight, as evidenced by the image of YFP-TAN1(28-33A)-YFP tan1 air9 seedlings in Figure 4B. I recommend either changing the labeling of these graphs to "Transverse Wall Angles," or to replace these measurements with those of the longitudinal walls.

RESPONSE: We further describe how the cell file rotation angles were measured in the materials and methods. In order to maintain consistency with previous reports on the tan1 air9 double mutant, we decided to be consistent with the method we used previously to collect and display the cell file rotation data.

Point 6. Please provide a more complete explanation how the ratio of YFP signals at the cortical division site and the cytoplasm (Figures 3K, 5E) was calculated. Was there any background subtraction performed? (This is relevant since Figure 7G indicates a substantial background signal in untransformed seedlings.) Which part of the cytoplasm was used for this comparison? Was the ratio based on average pixel intensities in the two areas?

RESPONSE: A more detailed description of how the ratio of YFP fluorescence at the division site and the cytosol was measured and calculated was added to the materials and methods section.

"The ratio of the division site versus cytosolic fluorescence intensity was determined by taking the median YFP fluorescence intensity from the center z stack of individual cells with PPBs or phragmoplasts. For each cell the median fluorescence intensity was measured for two cytosolic areas and the division site on each side of the cell using circles with areas of 0.875 μm2. The sum of the median intensity at the division site on each side was then divided by the sum of the median intensity of the two cytosolic areas to calculate the ratio of the division site versus

cytosolic fluorescence intensity. Fluorescence intensities were measured in FIJI."

Point 7. The subheading for the second part of the Results section is too strong has to be toned down. As mentioned before, it is not clear whether the effects of the 6-Ala mutation are solely cause by the disruption of TAN1-POK1 interactions. It is also not correct to say that this mutation "abrogates tan1 air9 rescue" since there is still a partial rescue of the mutant phenotypes possible.

RESPONSE: We toned down our language to state that rescue is reduced.

Point 8. The alanine linker scanning experiment in Supplementary Figure 2 does not appear to show all constructs tested. Please include the missing constructs or explain why they are not shown.

RESPONSE: Unfortunately we ran into some difficulties cloning sections 64-69 and 106-111 and they were not completed. We now state this in the Supplementary Figure 2 legend.

Reviewer #3:

The manuscript describes experiments related to the delineation of the cell division plane in plants. The primary question appears to be how TANGLED1 and POK1 lead to phragmoplast guidance during cytokinesis. The work builds on a previous discovery that tan1 shows an enhanced phenotype in the air9 background. The work further addresses prior discoveries about TANGLED1 domain function related to POK1 localization or activity.

The figures provide a reasonable level of detail to conclude that POK1 has an interaction with TAN1 through the first 132 aa in an area 30 aa from the N-terminus. Further data indicate that this interaction is only partly responsible for the action of either protein and that these actions require AIR9. The phenotypes are manifest during, or downstream of, cell division (mitotis).

Point 1. There is a lot of careful analysis of root phenotypes and the localizations are reasonable, though not entirely convincing in some cases (~Fig 3). The text of the manuscript is extremely difficult to parse, both in its organization and in many of the frames drawn around the experiments prior to their description. Moreover, judging the overall impact of the discoveries is clouded significantly by the writing. There are general issues with the characterization of the materials, most notably the reliance on constitutive promoters for nearly all of the work characterizing genes and domains that have roles in the timing and organization of cell division.

RESPONSE: Thank you for these insightful comments. We removed pKN:TAN1-YFP data from this manuscript to better focus our discussion on POK1, TAN1, and AIR9 interaction. We added new localization data that strengthens our conclusions. We used the TAN1 native promoter in CFP-TAN1 and CFP-TAN1(28-33A) plasmids to examine YFP-POK1 and TAN1 localization in the tan1 air9 double mutant. Although we don't include the data here, we demonstrate that TAN1 driven by its native promoter fully rescues the tan1 air9 double mutant here (https://doi.org/10.1101/2021.04.30.442137).

We introduced the microtubule marker, UBQ10:mScarlet-MAP4, into our YFP-POK1 tan1 air9 double mutants and single mutants. By doing so we were able to determine that YFP-POK1 is lost from the division site during metaphase. We also generated tan1 air9 lines that express the microtubule marker UBQ10:mScarlet-MAP4, pPOK1:YFP-POK1, and pTAN:CFP-TAN1 or pTAN:CFP-TAN1(28-33A) to better determine how disrupting POK1-TAN1 interaction affects their localization to the division site.

Point 2. The manuscript seems to be written with regard to the order in which the experiments were done, and not with regard to the importance or relevance of the biological hypotheses. From a reader's perspective, I want to understand how TAN1, POK1, and AIR9 function either independently and/or dependently to affect a correct cell division. To do that, I want the hypothesized mechanisms by which they work to be detailed in the introduction from peer-reviewed works so that I understand what biological mechanisms are under test or what observations are being sought in light of those hypotheses.

RESPONSE: We apologize for writing unclearly. We added additional information about the relationship between POK1, POK2, and TAN1 to the introduction to better describe why we are interested in studying POK1-TAN1 interaction. We describe how the pok1pok2 double mutant has phragmoplast defects and fail to maintain TAN1 at the division site. We clarify in the discussion that there currently is no information about how AIR9 and POK1 may

interact. However our data shows that POK1 localizes to the division site in the tan1 single mutant, which suggests that either AIR9 or an AIR9 interacting protein plays a role in POK1 maintenance at the division site.

Point 3. Having an unambiguous understanding of the single mutant phenotypes, the double mutant phenotypes, and the degree to which the native-promoter TAN1 or POK1 constructs quantitatively rescue the phenotypes being described would be an important start. If the goal for much of the study is to understand when and how (by what domains) the hypothesized interaction between TAN1 and POK1 lead to correct phragmoplast alignment, and how this involves AIR9, then frame the manuscript around those intentions. Seeing that the first 132 bp of TAN1 provide a level of rescue for the tan1 air9 double mutant is only interesting if it leads to a better informed mechanistic model for how the full-length proteins act in the wild type plant. Using that domain to probe for interactions with POK1 is fine, but the important result is knowing if the full length TAN1, without the domain that was found to act in the POK1 interaction, also does not bind POK1 (I could not find those data) and what the results are for rescuing phenotypes (different than the set up looking at the first 132 aa). In the end, I am not really certain what I can take away from the discoveries other than there is at least one interaction with POK1 at the N-terminal region of TAN1.

RESPONSE: Thank you, we now show in Supplementary Figure 3 that TAN1(28-33) does not interact with POK1 in the yeast-two-hybrid system. Using a microtubule marker, we quantitatively analyze how POK1 localizes to the division site in tan1 and air9 single mutants (Figure 1, Table 1). We also demonstrate that TAN1 driven by its native promoter, including the pTAN:CFP-TAN1 construct, fully rescues the tan1 air9 double mutant in a manuscript now posted on bioRxiv (https://doi.org/10.1101/2021.04.30.442137).

Point 4. The discussion section starts out as a re-litigation of the results section. Again, all of the interrelated details being rehashed here have little meaning out of the context of the biological hypotheses you set out test for how these proteins are acting in cell division. Instead of leaping into a pile of results, please take this space to redevelop why you are asking these questions and how the answers you have gotten either fill in major gaps or undermine what the field previously hypothesized as mechanism. Otherwise, these interesting observations look more like incremental steps rather than important discoveries.

RESPONSE: We clarify the specific questions we are answering and how they contribute to our understanding of division plane orientation. We significantly revised our introduction and discussion to include more relevant information about POK1 and amino acids 1-132 of TAN1, as well as other proteins that contribute to division plane positioning.

Point 5. The number of qualifiers in the first, very long paragraph of the discussion section is honest, but it also belies a missing element of the data interpretation. I would never fault any investigator for counting and then honestly providing the experimental counts. In this case, however, there are important biological questions about why you are not seeing a yes or no answer with many of the localizations and phenotypes. For example, the fraction of cells showing POK1 localization at the phragmoplast midline or the telophase division site, but apparently not both in the same cell, is not complete - why? And is the same cell only one or the other? I do not see how this comes about from a 'mutually dependent' set of interactions (second paragraph). Are these cases arising more probably because of sequentially dependent events that happen only sometimes in the rescued lines, or because the constructs are being expressed under a 35S promoter to different amounts, or is this because the interactions are on the cusp of what is kinetically possible for getting the gene products to hold together? Showing that the localizations are not the same as the control is great, but given what is being tested here, understanding why these localizations are partially affected and not blocked seems important to the interpretation. In summary, I believe the central findings of this work are interesting to the area of plant biology attempting to piece together how the known genes in this system work together to divide the plant cell correctly. There are some important issues to deal with regarding the construction of the manuscript, the absence of baseline control data, and specific experiments meant to reinforce claims about localizations.

RESPONSE: Thank you for this comment. We substantially revised our conclusions of YFP-POK1 localization in the tan1 air9 double mutant based on new data obtained from plants with a microtubule marker, now Figure 6 and Supplementary Figure 6. We also include detailed cell counts describing POK1 localization in wild-type, tan1, air9, and tan1 air9 plants in Table 2.

Point 6. Introduction: TANGLED1 (TAN1, AT3G05330) was the first identified protein that localizes to the plant division site throughout mitosis and cytokinesis (Walker et al., 2007). First protein to localize, or first protein identified in the division process?

RESPONSE: Reworded so it is clear that TAN1 was the first protein identified to localize to the division site in plants. "TANGLED1 (TAN1, AT3G05330) was the first protein identified to localize to the plant division site throughout mitosis and cytokinesis (Walker et al., 2007)."

Point 7. The introduction references a paper that has not been accepted in a peer reviewed journal where many of those referenced results are quite interesting, but not essential to the story here. From the perspective of this reviewer (and subject to Plant Cell policy), those observations should more likely be cited at this time as unpublished data or left out of the introduction at this time.

RESPONSE: Thank you for this comment. It is our understanding that Plant Cell prefers a specific reference to the material, so that the reviewer is able to judge for him/her-self rather than use the phrase (unpublished data).

Point 8. The penultimate introductory paragraph introduces information but does not make a coherent point. What is the argument being made and the intention with this paragraph (107-129). I am also unclear about the definition of a 'cell file rotation' phenotype (112) as there is a difference between root twisting, cells growing at an angle to the plant growth axis, and cells showing shifts between cell files.

RESPONSE: We have substantially altered the introduction to more clearly articulate the specific scientific questions we are addressing. We found that presenting the pKNOLLE work as its own topic made discussion of TAN-POK1-AIR9 interactions in this manuscript more focused. This data can be found here: (https://doi.org/10.1101/2021.04.30.442137).

Point 9. Results section: Related to comments above, why is this a constitutive 35s and not a native promoter experiment to ask about rescue? Since the protein is supposed to function at a very specific time in mitosis, and the protein is speculated to work in a complex where stochiometry could be important, it is surprising that the gene and gene fragments are not being driven by the native promoter to at least deal with the timing.

RESPONSE: Thank you for this comment. We agree that native promoter fusions are useful. In addition, we showed that p35S-TAN1-YFP rescued the tan1 air9 double mutant (Mir et al. 2018). Although we demonstrated that the 35S promoter construct rescues the phenotype, we have further shown that native promoter also rescues the tan1 air9 double mutant whether it has an N- or C- terminal fluorescent protein fusion (Mills and Rasmussen 2022). We used native promoter pTAN1:CFP-TAN1 or pTAN1-CFP-TAN1(28-33A) together with YFP-POK1 and UBQ10:mScarlet-MAP4 in the tan1 air9 double mutant to unravel their localization patterns .

Point 10. It is never explained here why the first 132 AA of the protein are used for these experiments relative to any other part of the protein. Even if there are experiments in a prior paper laying out the rationale, the reliance on this construct requires some level or explanation - even if it was just chance to find it. To what degree does this construct differ from the full length TAN and how much of the protein does this constitute? Is this a known domain or is this just a random site?

RESPONSE: Clarified importance of region I of TAN1 in the introduction: "TAN1 is an intrinsically disordered protein that can be divided into 5 conserved regions based on alignments of amino acid similarity across plant species. Region I which covers the first ~130 amino acids of TAN1 is the most highly conserved. This region has also been shown to mediate interactions between TAN1 and a number of other proteins including POK1 (Müller et al., 2006). Region I is also responsible for mediating TAN1 localization to the division site during telophase in Arabidopsis (Rasmussen et al., 2011)."

Point 11. You need to state clearly what you are testing with regard to comparisons and provide the rationale for using a statistical test that looks at the difference in variance as opposed to the difference in mean values. If you are asking whether the angle of 'cell file rotation' is the same or different between lines, I want to know exactly what is being measured to create that angular metric and you need to state that you are testing the significance that the angle varies to a different extent. Furthermore - this is a place where the rescue should be compared to the degree of rescue from the full-length native-promoter TAN construct or where it should be explained why that is not important

here. Levene's test is going to show a marked sensitivity to n number making the claims in 1E for PPB, and to a lesser extent, Phragmoplast, more difficult to judge.

RESPONSE: Thank you for this comment. We have substantially clarified several sections including materials and methods. "F-test was used to compare normally distributed variances (PPB and phragmoplast angles) and Levene's test was used to compare non-normally distributed variances (cell file angle measurements)."

Point 12. I do not see any developed connection between the ideas in this sentence or the evident reasoning: 164 Since p35S:TAN11-132-YFP effectively rescued the tan1 air9 double mutant, we focused on 165 further understanding how TAN1 functioned by disrupting its ability to interact with the kinesin POK1.

RESPONSE: Added additional information about how POK1/2 are involved in TAN1 maintenance at the division site: "pok1 pok2 double mutants also fail to maintain TAN1 at the division site. TAN1 is recruited to the PPB but is lost from the division site upon entry into metaphase in the double mutant (Lipka et al., 2014). This suggests that TAN1 maintenance at the division site after metaphase is dependent on POK, but TAN1 is not required for POK1 localization."

Point 13. The apparent focus of the paragraph and the experiment is given with the hypothesis in lines 174-176. That would be important to know at the beginning of the paragraph when trying to sort out what the goal will be. Furthermore in 167-168, it took quite some time to work out how a scanning alanine mutagenesis produced 17 constructs from 6 amino acid positions. Please explain the experiments in the text and be clear about their intent.

RESPONSE: We clarify that we were unable to clone two alanine scanning substitutions at positions 64-69 and 106-111 in the figure legend.

Point 14. The word 'systematically' does not infer permutations or combinations where scanning typically means one at a time 'scanning' through the protein and 'tiled' means overlapping. More related to the experiment, why were '6' amino acids chosen and what were the commonalities for the seven that did not show interaction in the two-hybrid assay? What is the reason you attribute to the loss of interaction - charge, structure, folding?

RESPONSE: 6 amino acids were chosen because it was the largest number of amino acids that we could reliably alter with less expensive primers. We clarified how we designed our alanine scanning constructs in the materials and methods section. We include the following sentences in the discussion on loss of interaction: "Here we hypothesize that amino acids 28-33 are essential for TAN1 and POK1 interaction in both yeast-two-hybrid and in Arabidopsis. In addition to several reports showing that TAN1 and POK1 interact using the yeast-two-hybrid system (Müller et al. 2006; Rasmussen et al. 2011), bimolecular fluorescence complementation has also been used to show TAN1-POK1 interactions in Arabidopsis protoplasts (Lipka et al. 2014). Alanine substitutions at positions 28-33 of TAN1 may disrupt TAN1-POK1 interaction through misfolding that blocks the POK1 interaction site or by affecting the amino acids that directly mediate POK1 binding. Regardless of the exact mechanism(s) of POK1-TAN1 physical interactions or the possibility that yeast-two-hybrid interactions do not reflect equivalent POK1-TAN1 physical interactions in Arabidopsis, we show that these TAN1 amino acids are involved in mediating TAN1 and POK1 localization to the division site."

Point 15. 189-200. It seems reasonable that the TAN alanine replacement fragment does not have the same ability to localize at the division site as the full length protein - based on the images provided. Taking the ratio of the fluorescence (done again at 216-217) at the presumed division site and the cytoplasmic fluorescence as a means of supporting the claim makes little sense to me here. Suppose that under the constitutive promoter, one cell or plant has twice as much expression as the other, and the same number of binding sites for the protein at the presumptive division site. Your ratio of intensities (taking microscopy issues as being all equal) is then much more about the relative expression levels than the actual extent of localization when taken as a ratio. As the microscope characterization was not formally presented, it may also be the case that there are substantial issues with the values being assumed to represent cytoplasm and division site protein concentrations.

RESPONSE: Indeed, although we recognize the drawbacks mentioned here, using fluorescence intensity measurements was simply the best way we could measure relevant accumulation at a specific location. We more clearly discuss how fluorescence intensity measurements were taken in our updated materials and methods section. "The ratio of the division site versus cytosolic fluorescence intensity was determined by taking the median

YFP fluorescence intensity from the center z stack of individual cells with PPBs or phragmoplasts. For each cell the median fluorescence intensity was measured for two cytosolic areas and the division site on each side of the cell using circles with areas of 0.875 µm2. The sum of the median intensity at the division site on each side was then divided by the sum of the median intensity of the two cytosolic areas to calculate the ratio of the division site versus cytosolic fluorescence intensity. Fluorescence intensities were measured in FIJI. All plants used for this analysis were grown on the same day and imaged using identical conditions.

Point 16. There seems to be a big leap between the supposition drawn on lines 199-201 (TAN1 localization is partly abrogated by absence of POK1) and the conclusion drawn at the beginning of the next paragraph (203-204) - (POK1 uses a direct interaction to recruit TAN to the division site). While the evidence presented is consistent with the model, I do not think it is clearly evident from the data in this paper at this point. And how this is resolved from the idea that it is not critical when TAN1 is 'already at the division site' is left unexplained at the end of the paragraph.

RESPONSE: We have substantially revised this part of the manuscript.

Point 17. Where is it shown that the full-length TAN1 with the 6-alanine replacement does not bind to POK1? I think this is assumed because of the TAN1-132 result, but I do not see any evidence in the manuscript that this is actually the case. I might be missing this somewhere.

RESPONSE: Thank you for the suggestion. We added supplementary figure 3, which shows that full length TAN1(28-33A) does not interact with POK1 by yeast two-hybrid.

Point 18. This is most confusing in the way the manuscript has been written, but I think it is interesting that the POK1 localization is mostly different in the double mutant when compared to the single mutants. Perhaps not surprising, given the double mutant phenotype, but interesting.

RESPONSE: Including a microtubule marker in our transgenic lines has allowed us to collect more complete localization data.

Point 19. The motivation for the use of a mitotic promoter is developed with some assumptions, but leaves out two key issues. First, why is there no native-promoter TAN1 line complementing the tan1 air9 mutant throughout this work and, given the apparent incomplete rescue with the current full-length construct, why is there not a version without the fluorescent protein?

RESPONSE: We removed the KNOLLE promoter data to focus more on how division site protein interactions. We collected data demonstrating that TAN1 driven by its native promoter (including the pTAN:CFP-TAN1 lines presented in this manuscript) fully rescues the tan1 air9 double mutant. This is now a separate manuscript available on bioRxiv where we more carefully discuss the significance of the KNOLLE driven TAN1 rescue (https://doi.org/10.1101/2021.04.30.442137).

Point 20. It seems that either of these properties of the transgene being assessed for rescue could be leading to malfunction. Importantly, if either issue is contributing to phenotype, it would alter the findings for most but not all of the results. Regarding the motivation for using the mitotic promoter, the authors assume that this will allow them to dissociate mitotic from interphase functions. However, they indicate in the introduction (I think) that there are a number of genes that affect only mitotic function that still give rise to the post-mitotic phenotypes (e.g., organ twisting) that they want to address. Whether this is an issue with trying to introduce the experiment (writing), or with the logic of the experiment is not clear. The result is interesting and rather direct, but made very confusing by the way it was presented.

RESPONSE: We have substantially revised the manuscript, based on helpful suggestions from you and other reviewers, and based on exciting new data.

TPC2022-RA-00398 1st Editorial decision – *revision requested* June 27, 2022

We have received reviews of your manuscript entitled "The localization of PHRAGMOPLAST ORIENTING KINESIN1 at the division site depends on two microtubule binding proteins TANGLED1 and AUXIN-INDUCED-IN-ROOT-CULTURES9 in Arabidopsis." We are very sorry that there was a significant delay in this decision. On the basis of

the advice received, the board of reviewing editors would like to accept your manuscript for publication in The Plant Cell. This acceptance is contingent on revision based on the comments of our reviewers. In particular, please consider the following:

1. Please address reviewer 1's major and minor concerns.

2. Based on the initial review of the manuscript, there was enthusiasm for the study and reviewer 1 is still enthusiastic. However, the new reviewer 2 was less enthusiastic. We ask that you respond to aspects of their review that do not speak to the novelty of the study.

-- Reviewer comments:

TPC2022-RA-00398R1 1st Revision received July 26, 2022

Reviewer comments on previous submission and **author responses**:

Reviewer #1

How vascular plant cells maintain planes of division is an important and long-standing research topic because of its relevance during pattern formation and growth. In the current manuscript, the authors investigated aspects of the interaction between TAN and POK1. Both proteins are located at the site of division during cell division, and phenotypes of maize and Arabidopsis mutants assign these proteins roles in phragmoplast guidance. Previous work in Arabidopsis showed that recruitment of TAN1 to the PPB was not dependent on POKs, while its maintenance at the division site was.

In the manuscript, the authors examined POK1 localization in air9tan1 mutants. The absence of POK1 at the division site after PPB disassembly suggested a role for TAN1 and AIR9 in maintaining POK1. To better understand how TAN1 interacts with POK1, the authors used the TAN1 N-terminal 132aa fragment, which is necessary and sufficient for POK1 binding in yeast two-hybrid assays, as identified in a previous publication. Despite the absence of the fragment from the division site up to telophase, air9tan1 PPB and phragmoplast defects could be rescued. Can TAN1 protein levels be too low for visualization at PPB? The authors hypothesized that phragmoplast rescue might be achieved due to the ability of TAN1(1-132) to interact with POK1, although they did not investigate the localization of POK1 in the respective plant lineage.

Using alanine substitutions, they set out to identify domains within TAN(1-132) relevant to POK1 interaction and selected a conserved domain for further study. The alanine substitution mutant TAN(1-132)28-33A appeared to be impaired in its ability to associate with the division site and rescue the mutant phenotype, presumably caused by reduced POK1 binding.

The authors argued that introducing the alanine substitutions into full-length TAN1 should abolish TAN1's ability to bind POK and not salvage the air9tan1 phenotype. Contrary to the hypothesis, POK1 accumulated at the site of division, albeit at a reduced level, similar to the TAN1(28-33A) transgene itself. The result is puzzling and suggests that additional proteins, likely MAPs/ and/or other division site proteins, work redundantly with TAN1 and AIR9. This explanation is not entirely consistent with the absence of POK1 in air9tan1 and its phenotype resembling pok1 pok2.

The results are of great interest, although I disagree with some interpretations. Furthermore, it is not clear to me how the authors envision the interaction of TAN1 and POK1 in phragmoplast guidance.

Point 1. Expression of TAN1(1-132) partially rescues the phenotype of the PPB mutant, although it localizes only in telophase at the division site. If I understand correctly, the authors speculate that phragmoplast guidance relies on the recruitment of POK1 to the site of division during telophase via TAN1(1-132). To confirm this, the localization of POK1 in plants expressing TAN1(1-132) is necessary. This should clarify whether there is a second recruitment phase of POK1 during telophase as suggested by the authors. Alternatively, POK1 could be present at the site of division throughout mitosis, but regulatory events could control TAN1 and POK1 interaction. In any case, it would be

important to know if this fragment is sufficient to recruit POK1 to the cleavage site and to understand phenotype rescue.

RESPONSE: Thank you for this comment, we agree that understanding how POK1 localizes in tan1 air9 mutants expressing TAN1(1-132) and in TAN1(1-132)-28-33A is a critical next step. For this manuscript, we decided to use the full-length construct instead of TAN1(1-132). Our reasoning was that it was a more targeted way to assess TAN1-POK1 interactions, with less potential for disrupting other interactions. Further, as discussed in point 2 below, the imaging is challenging for TAN1(1-132) due to the strong nuclear accumulation.

Point 2. Figure 3: The examples of telophase location of TAN1(1-132) and TAN(1-132)28-33A at the division site look like late phragmoplast midzone locations to me. Can you provide better examples? Can you rule out that TAN(1-132) and/or POK1 only reach the site of division during cell plate fusion via the phragmoplast in these plants?

RESPONSE: We appreciate this comment. We did not look at POK1 in these lines, although that is something that we plan to do in the future. The imaging for these lines is difficult, so we cannot provide better examples. We looked very closely at the localization of TAN1(1-132) and TAN1(132)28-33A specifically at the division site versus the phragmoplast midzone, and ruled out that TAN1(1-132) only reaches the division site during late stages (cell plate fusion). In addition, these results are consistent with earlier time-lapse imaging (Rasmussen et al. 2011) showing that TAN1(1-132) accumulates at the division site before the phragmoplast contacts at that location (please look at the left hand side of the cell to see the TAN1(1-132) accumulation at 30 and 42 minutes.

Point 3. In TAN1(28-33A)-expressing mutant plants, both the TAN1 transgene and POK1 localize correctly, although not in all mitotic cells. Therefore, the authors speculate that the reduced rescue ability stems from cells where POK1 failed to associate with the division site and therefore the phragmoplast is not guided. They could test this hypothesis by live imaging cytokinetic cells expressing TAN1(28-33A) in the presence and absence of POK1?

RESPONSE: Thank you for suggesting this valuable experiment. We plan on doing it, but it is outside the scope of the current manuscript.

Point 4. The TAN(28-33A) mutant partially rescues the phenotype and co-localizes with POK1 at the division site. The authors speculate that other residents of the division's site may facilitate their recruitment, which explains the partial rescue. For example, IQD8 is mentioned as a potential POK1 If so, how do you explain that POK1 is missing from the division site in the *air9 tan1* mutant?

RESPONSE: We showed that POK1 localization is dependent on both TAN1 and AIR9 (Figure 1). Effectively, in the tan1 air9 double mutant expressing TAN1(28-33A), AIR9 is missing, and TAN1 is expressed, but unable to interact with POK1. Our hypothesis is that TAN1 and POK1 interactions are both direct and indirect. While direct interactions are disrupted in the tan1 air9 double mutant expressing TAN1(28-33A), we hypothesize that indirect interactions still occur, allowing partial POK1 retention at the division site. In other words, we hypothesize that both TAN1 and POK1 have additional interactors that stabilize their interaction at the division site. Evidence suggests that these interactions occur independently of amino acids 28-33 in TAN1.

Point 5. The authors use p35S driving TAN1(1-132) and TAN1(1-132)28-33A. How much transgene-silencing do you see in these populations?

RESPONSE: We had problems with transgene silencing in some of our lines (approx 1 in 5), but we had enough lines to focus on those that did not have transgene silencing.

Point 6. The display of frequencies of POK1 and TAN1 variants can be improved. For example, bar charts could be used to visualize localization frequencies in rescue lines and co-expression lines. A more visual approach would help gather the information more quickly and changes in frequency would be obvious. In my view, Table 1 is only of limited use for this purpose and could be moved to supplements.

RESPONSE: Thank you, we have updated our tables to bar charts and now include them in the figures. This greatly improves them.

Point 7. Line 145: Lipka et al. showed a similar frequency of colocalization of POK1 and PPB.

RESPONSE: Thank you, we have included that reference (now L141): In wild-type cells, YFP-POK1 colocalized with

PPBs in 71% of preprophase/prophase cells (n = 50/70 cells, 15 plants, Figure 1A) consistent with previous observations (Lipka et al., 2014, Schaefer et al., 2017).

Point 8. Line 208 and supplemental Figure 7: why not show the orientation of TAN1-132 and use boxes to indicate all regions replaced in alanine scanning mutagenesis?

RESPONSE: Thank you for this comment. Supplementary Figure 7 shows the conservation between species for amino acids 28-33A (as requested by previous reviewer 3), and it would become very cluttered if we boxed out all the substitutions. Please see Supplementary Figure2 for the boxed schematic.

Point 9. Line 257: Independence of TAN1 recruitment from PPB in prophase confirms similar results of Lipka et al.;

RESPONSE: Thank you, we have included that reference in both locations (now L141 and L264): In wild-type cells, YFP-POK1 colocalized with PPBs in 71% of preprophase/prophase cells (n = 50/70 cells, 15 plants, Figure 1A) consistent with previous observations (Lipka et al., 2014, Schaefer et al., 2017).

PPBs in *tan1 air9* mutants containing unaltered CFP-TAN1 and YFP-POK1 (Figure 6A, Table 2; Fisher's exact test, P-value = 0.0001, similar to (Lipka et al., 2014, Schaefer et al., 2017)).

Point 10. Line 258: The very mild tan1 mutant phenotype in Arabidopsis does not support the statement that defects in phragmoplast positioning could be specifically due to disruption of the TAN1-POK1 interaction or to a lower accumulation of TAN1 at the division site.

RESPONSE: Please note that these interactions only become important when AIR9 is missing also. In this context, interaction between TAN1 and POK1 and/or their accumulation become more relevant. We have updated the text to better reflect that. Defects in phragmoplast positioning may be due specifically to the disruption of TAN1-POK1 interaction in the tan1 air9 double mutant. Since AIR9 is missing, TAN1 and POK1 interactions may be more important. Alternatively, phragmoplast guidance defects are due to the lower accumulation of TAN1 at the division site that would normally be mediated by POK1 during telophase.

Point 11. Line 332: POK1 recruitment to the division site was delayed in trm678; I would substitute "disrupted" as it could be misconstrued as absent.

RESPONSE: Thank you for this comment, we have updated the manuscript to better reflect how POK1 recruitment is altered in the *trm6,7,8* triple mutant

"*trm678* mutants, which lacked well defined PPBs, had partially disrupted POK1 recruitment to the division site but only minor defects in division positioning (Schaefer et al., 2017). "

Point 12. Why did you use median intensity instead of mean intensity for the ratio of division site to cytosolic signal?

RESPONSE: We used a uniform sized circular ROI on the TAN1-YFP (and other TAN1 YFP fusion constructs) puncta at the division site. The median provides a better measurement of YFP intensity of the puncta rather than the mean due to the potential for a small area of background lacking YFP within the ROI becoming incorporated into the average.

Reviewer #4:

This manuscript describes protein interaction of TANGLED1 (TAN1) in relation to the division site localization. The authors previously demonstrated that TAN1 is divided to 5 regions: the region I (1-132a.a) interacts with POK1 and the region II (126-229a.a) is responsible for division site localization at the phragmoplast stage (Rasmussen et al. 2011). The authors also found that tan1 mutant in A. thaliana grows normally but tan1 air1 double mutant shows defects in orientation of cell division (Mir et al. 2018). The authors recently found TAN1 directly binds with microtubules in vitro (Martinez et al. 2020). In this paper, the authors show five amino acids (28th-33rd) in the region I mediate POK1-TAN1 interaction. In addition, the whole polypeptide excluding the region I recruits POK1 during metaphase and telophase (possible indirect effects). Mis-localized POK1 is recruited to the phragmoplast midzone as its family kinesin POK2.

Although the authors add many data after the previous study, the observed POK1 localization is easily expected by instable phragmoplast orientation shown in Mir et al. (2018). Determination of responsible amino acids for POK1 interaction is extension of two-hybrid analyses in Rasmussen et al. (2011). Unclear defects in whole TON1 with 5-aa mutation, compared with Region I expression, suggests that TON1 and POK1 interact in multiple ways, and current deletion analyses cannot address the mechanism of POK1 localization. In my impression, efforts of this work are not fruitful for elucidation of the mechanism of POK1 localization.

Point 1. The title stress dependency of AIR9 and TAN1 on POK1 localization, but the manuscript focuses TAN1 deletion and rescue studies. The present title does not match to the contents.

RESPONSE: Thank you for this comment. We believe this title most accurately reflects the main findings of the manuscript. Although more attention is focused on TAN1, we have carefully examined POK1 localization in tan1, air9 and tan1 air9 double mutants (Figure 1).

Point 2. In introduction, the authors describe there full-length TAN1 with mutated motif leads loss of POK1 and TAN1 (line 127-128), but CFP-TAN1 (28-33A) and YFP-POK1 is still maintained in 65% cells (line 277). It is difficult to say TAN1-POK1 interactions plays a critical role in stabilizing them together at the division site (line 287-288).

RESPONSE: We have modified this sentence to "Direct TAN1-POK1 interactions partially stabilize their continued localization at the division site".

Point 3. Changes in POK1 at the division sites during progression of cell division is difficult to understand from Table 1 and 2. The tables include localization types and cell cycle stages in vertical axis together. Please redraw the tables with cell cycle stages in the vertical axis and types of localization in the horizontal axis.

RESPONSE: Thank you for this suggestion, due to your suggestions together with Reviewer 1, we have converted the tables into bar graphs as part of other figures to make them more clear, please see above.

Point 4. Journal name in the reference list is missing in Clough and Bent (1999), Kirik et al. (2007), Rasmussen et al. (2011), Schaefer et al. (2017). Page number is missing in Martinez et al. (2020), Rasmussen and Bellinger (2018), Wu et al. (2018).

RESPONSE: We have updated these references.

TPC2022-RA-00398R1 2nd Editorial decision – *acceptance pending* August 8, 2022

We are pleased to inform you that your paper entitled "The localization of PHRAGMOPLAST ORIENTING KINESIN1 at the division site depends on two microtubule binding proteins TANGLED1 and AUXIN-INDUCED-IN-ROOT-CULTURES9 in Arabidopsis." has been accepted for publication in The Plant Cell, pending a final editorial review by a science editor. At this stage, your manuscript will be evaluated by a science editor with respect to its presentation of scientific content, compliance with journal policies, and presentation for a broad readership.

Final acceptance from Science Editor August 22, 2022
